# Embodied artificial agents for understanding human social cognition

**DOI:** 10.1098/rstb.2015.0375

**Published:** 2016-05-05

**Authors:** Agnieszka Wykowska, Thierry Chaminade, Gordon Cheng

**Affiliations:** 1 Engineering Psychology, Division of Human Work Sciences, Luleå University of Technology, Luleå 97187, Sweden; 2 Technische Universität München, Institute for Cognitive Systems, Arcisstraße 21, 80333 München, Germany; 3 Institut de Neurosciences de la Timone, Aix Marseille University—CNRS, Marseille 13005, France

**Keywords:** artificial agents, social cognition, humanoid robots, social interaction, human–robot interaction

## Abstract

In this paper, we propose that experimental protocols involving artificial agents, in particular the embodied humanoid robots, provide insightful information regarding social cognitive mechanisms in the human brain. Using artificial agents allows for manipulation and control of various parameters of behaviour, appearance and expressiveness in one of the interaction partners (the artificial agent), and for examining effect of these parameters on the other interaction partner (the human). At the same time, using artificial agents means introducing the presence of artificial, yet human-like, systems into the human social sphere. This allows for testing in a controlled, but ecologically valid, manner human fundamental mechanisms of social cognition both at the behavioural and at the neural level. This paper will review existing literature that reports studies in which artificial embodied agents have been used to study social cognition and will address the question of whether various mechanisms of social cognition (ranging from lower- to higher-order cognitive processes) are evoked by artificial agents to the same extent as by natural agents, humans in particular. Increasing the understanding of how behavioural and neural mechanisms of social cognition respond to artificial anthropomorphic agents provides empirical answers to the conundrum ‘What is a social agent?’

## 1. Introduction

Numerous cognitive mechanisms are involved in human social interactions, illustrating the high social competence of our species. The mechanisms of social cognition are often subtle and implicit [1]. The second-person approach of social interaction [1] stresses the importance of natural social interaction protocols for understanding the way the human brain uses these mechanisms of social cognition. The challenge with using second-person perspective, however, is that the experimental protocols lose some of the experimental control offered by more traditional observational approaches. In this context, we postulate that using artificial agents, in particular embodied real-size humanoid robots such as CB [2], to study human social cognition offers a perfect compromise between ecological validity and experimental control. Artificial agents allow for manipulation of various characteristics of appearance and/or behaviour and for examining what impact those manipulations have on the mechanisms of human social cognition [3]. In support of this idea, Sciutti *et al.* [4] argued that using humanoid robots is beneficial for examining how observers understand intentions from movement patterns of the observed agents thanks to the ‘modularity of the control’ [4, p. 3]. Modularity of control means that it is possible to decompose precisely and reproducibly robot movements into elements, an impossible endeavour for a human, and to examine separately the contribution of each of the elements to how observers understand intentions.

Importantly, while allowing for experimental control and manipulation, artificial agents offer certain degrees of social presence and realism, in contrast to more abstract or simplified stimuli such as schematic faces.

Artificial (embodied) agents can be used in the study of social cognition in a twofold manner. They can play a role of ‘stimuli’, or agents that participants observe/interact with; or they can serve as embodied models of social cognition. In the first case, embodiment is critical for studying social cognition due to the fact that real-time interactive scenarios with an embodied agent are crucial for mechanisms of human social cognition [5–7], while in the second case, serving as models of social cognition in a naturalistic social environment, they also need to be embodied. This paper will focus only on the first case: artificial embodied agents used as ‘stimuli’ in studying social cognition.

**Figure 1.**
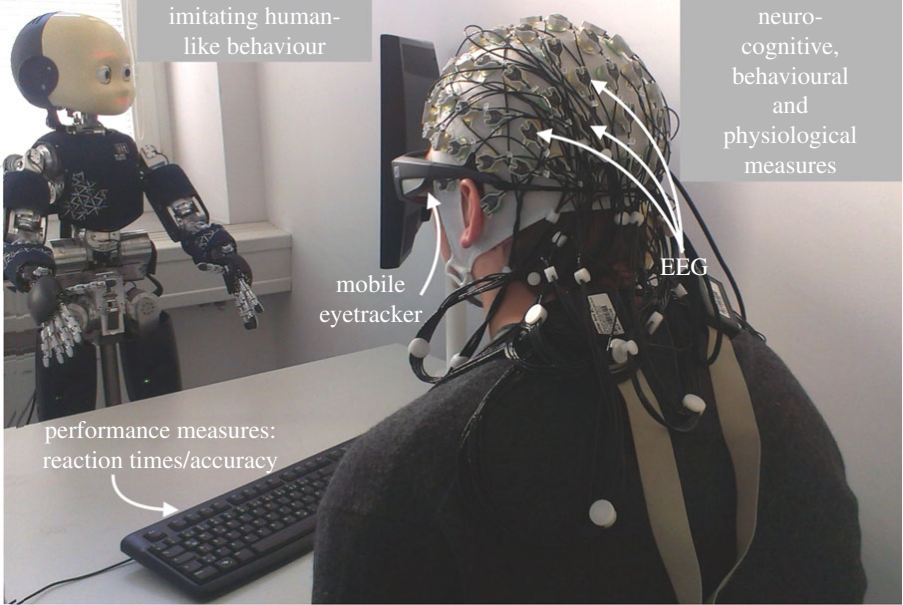
Illustration of an example experimental set-up in which a human interacts with a humanoid robot iCub [8], while behavioural, neural and physiological measures are taken to examine the human social cognition. (Online version in colour.)

The paper will review several behavioural and neural mechanisms of social cognition examined with the use of artificial agents and humanoid robots in particular (figure 1). First, in §2, low-level mechanisms of social cognition (such as motor and perceptual resonance) will be reviewed in the context of whether they are evoked by interactions with artificial agents. In §§3–5, the paper will describe mechanisms gradually increasing in hierarchy, up to the level of higher-order cognition, such as mentalizing or adopting the intentional stance. Most importantly, the paper will attempt to answer the question: can we be ‘social’ with agents that are of different ‘kind’ than our own species, and in particular, if they are not a natural kind but man-made artefacts. The paper will conclude in §6 by summarizing the benefits of using artificial agents for the study of social cognition.

## 2. Action–perception coupling

One of the key mechanisms of social cognition is the ability to understand other agents' actions. Understanding others' actions is based—at least partially—on the activation of action representation by the observer [9,10]. Therefore, perception and action systems are tightly coupled to allow for processing of perceptual information and motor control in an integrative manner. This has been postulated by theories inspired by the ideomotor perspective [11–13]. For example, proponents of the ‘theory of event coding’ or the general ‘common-code’ perspective [13–15] claim that action and perception share a common representational code. The discovery of mirror neurons [10] tagged a common neural mechanism for action and perception domains and provided evidence for the common coding hypothesis [9,16–18], which posits that observing an action automatically triggers activation of action execution representations. Interestingly, mirror neurons are also active when the meaning of an action can be inferred from sounds [9] or other hints [19]. These findings have been taken to support the idea that the mirror neuron system plays a functional role for action understanding [20]. Some authors have proposed that the mirror neuron system is responsible not only for action understanding, but also for imitative learning [21] and may even provide a basis for communication and language acquisition [22]. Because of common coding, action observation impacts activity in the motor system of the observer (*motor resonance*).

### (a) Motor resonance

A consequence of motor resonance is that seeing an action hinders the execution of a different action (motor interference) and facilitates the execution of the same one (automatic imitation). This property was used in two series of behavioural experiments using humanoid robots to investigate factors influencing motor resonance. In one series of experiments, participants performed continuous arm movements in one direction while observing another agent performing continuous arm movements in the same (congruent) or an orthogonal (incongruent) direction. Because of motor interference the movement was less stable in the latter condition, so that the ratio between movement variance in the incongruent and congruent conditions was used as a marker of motor resonance. Originally, this paradigm supported an absence of motor interference when the observed agent was a robotic arm [23]. Using the humanoid robot DB instead of an industrial robotic arm, the same paradigm indicated that a humanoid robot actually triggered a motor interference effect [24], though reduced compared with a human. In a follow-up study, Chaminade & Cheng [3] reported that the interference effect disappeared if the humanoid body was hidden by a cloth, therefore reproducing the original finding.

Another series of experiments used a hand-opening paradigm [25–28]. Participants had to perform a hand-opening or -closing gesture and the onset of the movement was cued by the observation of a human hand or robotic claw opening or closing. Automatic imitation was evidenced by an increased reaction time when the observed and executed gestures were incongruent compared to congruent, and was larger for human than for the robotic stimuli [25]. Manipulating participants' beliefs about the nature of the agent controlling the movement, showing a human hand while pretending it was a robot control, did not result in top-down influence on the interference effect [26]. By contrast, repeated exposure to the robot in the congruent condition eliminated the increase of this effect for humans [27].

### (b) Action-related bias in perceptual selection

Wykowska and co-workers [29–32] investigated how action planning influences *early* perceptual processes in the visual domain. A series of experiments consisted of a visual search task for size- or luminance-defined pop-out targets combined with two actions: grasping and pointing. The paradigm created two congruent perception–action pairs according to ideomotor theories [11,12]: size-grasping and luminance-pointing. The results showed congruency effects in behaviour [29–31], with better search performance when size was coupled with grasping (as compared to pointing) and when luminance was combined with pointing (relative to grasping), as well as in event-related potentials (ERP) of the electroencephalogram (EEG) [32], with action-related modulation of early attention-related ERP components. These results are in line with previous findings of Fagioli *et al.* [33] in which processing of perceptual dimensions of size and location was biased with respect to pointing and reaching actions. Interestingly, in a later study [34], the authors showed that mere observation of an action performed by others (without execution of the action) is sufficient to elicit an effect of action-related bias on perceptual processing.

The congruency effects observed in [29–32] as well as in [33,34] were replicated when robot hands were used as stimuli [35]. Participants were also asked to perform two tasks—a perceptual task (a visual search task for a target defined by size or luminance), and a movement task—grasping or pointing. Similarly to [29–34], the design created two action–perception congruent pairs: size was coupled with grasping while luminance was coupled with pointing. The to-be performed actions were signalled either by robot-like or human-like hand stimuli. Action–perception congruency effects were observed both with robotic hands as well as human hands, which is in line with previous results [24].

A perceptual phenomenon related to motor resonance is perceptual resonance, the effect of the action people are producing on their perception of others' actions [36]. For example, if participants have to judge the weight of boxes lifted by other people while lifting boxes themselves, the observed weights are under- or over-estimated depending on the weight of the participant's own box [37]. These effects were preserved when the humanoid robot iCub [8] was performing the lifting actions [38,39].

### (c) Motor resonance network

Neuroimaging provides tools to investigate how parietal and premotor areas of the motor resonance network, that correspond physiologically to the human mirror system, respond to robotic actions and, in turn, what the features of visual stimuli are that affect their response. Interestingly, an fMRI experiment in awake macaque monkeys demonstrated a somehow reduced, but still large, response of an anterior premotor area buried in the arcuate sulcus, and supposedly homologous to the anterior part of Broca's area in humans, to a robotic hand performing a grasping movement compared with a human hand [40]. This clearly shows that the quest for mirror system responses to humanoid robots in human inferior frontal and parietal cortices is warranted. Historically, the first neuroimaging experiment using positron emission tomography (PET) reported increased response for the human, compared with the robot, in the left premotor cortex and concluded that ‘the human premotor cortex is “mirror” only for biological actions' [41]. This has been contradicted by subsequent fMRI studies, and is likely to have its explanations either in the technique used, PET reducing the number of conditions and contrasts that can be run, or in the robotic device used. Subsequent fMRI experiments using a similar stimulus (robotic hand grasping an object) found parietal and premotor response to both human and robotic stimuli [42], and an increase in the response of dorsal and ventral premotor as well as parietal cortices in the left hemisphere. Similarly, a Lego robot dancing was associated with increased response in inferior parietal lobules bilaterally [43]. By contrast, an electrophysiological marker of motor resonance, the mü rhythm suppression, was shown to be reduced when observing a robot's *versus* a human's action [44]. Interestingly in the two fMRI studies, participants were explicitly required to pay attention to the action being depicted, but only implicitly in the EEG experiment, in which they were to count the number of times the movie depicting the action stopped. Another result indeed suggests that motor resonance in inferior frontal cortices is sensitive to task demands [45]: response in bilateral Brodmann area 45 was significantly more increased when judging the intention behind the observed action (in that case, an emotion) relative to a more superficial feature of the action (the quantity of movement) for robot compared with human actions. This was interpreted as an increased reliance on resonance when explicitly processing the robot's movements as an intentional action compared with mere artefact displacements (see §4).

Altogether, this line of research suggests that motor resonance responds to human-like artificial agents, albeit this effect being reduced compared with real humans in some cases [24,45]. In other cases [38,39] the motor/perceptual resonance effect was at the same level for a humanoid robot as for a human. Thus, whether the motor/perceptual resonance effect is reduced when observing a robot as compared to observing a human might depend on the type of robot, its kinematic profile [46] or the type of task being performed. fMRI results not only confirmed a reduction of activity in an area associated with motor resonance, but also demonstrated that this reduction could be reversed by explicitly instructing the participant to process robot stimuli as ‘actions', therefore demonstrating a complex interplay between processing of sensory information and internal state of mind in motor resonance towards humanoid robots. In sum, the existing body of literature related to the low-level mechanism of social cognition, namely the motor- and perceptual resonance, suggests that observed actions of human-like artificial agents can indeed evoke resonance mechanisms. This suggests that low-level resonance mechanisms are not completely sensitive to whether the interacting agent is of a natural or artificial kind, as long as the observed actions can be mapped to one's own motor repertoire [46]. As perceptual and motor resonance are among the fundamental mechanisms of social attunement in interactions, it seems that fundamental (and implicit) level of attunement is possible also with artificial agents. But is it the same also for other mechanisms of social cognition, such as perceptual processing and higher-order cognition?

## 3. Perceptual processing

### (a) Early perceptual processing

Observation of actions executed by a robot, whether a full body robot dancing [43,47], a humanoid torso depicting emotions [45] or a simple robotic hand and arm grasping an object [41] is systematically associated with increased response in early visual areas of the occipital cortex compared with observing a human, including areas supposedly responsive to human form such as the fusiform face area [45]. Interestingly, this fits with the predictive coding account of visual processing, in which part of the feed-forward information is an error of the local prediction [48]. This error is larger for robots because of their imperfect human-like static and dynamic visual features. Interestingly, this increased response in early visual areas was no longer present in integrative areas, such as the temporo-parietal junction [45], that might actually respond to congruence between stimuli dimensions rather than the dimensions themselves [43]. The congruence between form and motion in particular could be the source of the hypothesized Uncanny Valley phenomenon [49], which states that an embodiment that resembles a human but in an imperfect manner causes negative emotional response. By comparing brain response to an android (human-like robot) to that of the human after whom the android was modelled, or of the corresponding humanoid (mechanical robot), Saygin, Chaminade and colleagues [50] reported an increased repetition suppression effect to the android in visual areas and regions of the action–perception system associated with attention (intraparietal sulcus). The actions of the android used in this experiment presented a clear mismatch between human-like appearance and robotic-like movements, putatively triggering an increased error signal in visual areas associated with these dimensions of the stimulus (in particular in the lateral occipital cortex) that induced increase in attentional resources recruited to resolve this discrepancy.

### (b) Joint attention

Another fundamental perceptual mechanism of social cognition is joint attention: the triadic coordination between at least two individuals and their focus of attention, wherein the individuals attend to each other and also to the content of their attentional focus, thus sharing attention [51,52]. A large body of evidence has demonstrated that humans attend to where others attend (joint attention), e.g. [53,54]. Joint attention can be established through, for example, following others' gaze direction. Capacity for joint attention is an essential component of the ability to infer mental states of others, and helps establishing a common social context, e.g. [51,54]. Joint attention has been extensively studied using the gaze-cueing paradigm (e.g. [55,56]) in which a face is typically presented centrally prior to the onset of a target in the periphery. Subsequently, the eyes are directed towards one of the sides of the visual field—a potential target position. In a typical gaze-cueing study, processing of the target (detection, localization, or discrimination) is facilitated when the gaze direction and target position coincide (*validly* cued targets), relative to when the gaze is directed elsewhere (*invalidly* cued targets); the difference in performance towards validly cued versus invalidly cued targets constitutes the *gaze-cueing effect*. The gaze-cueing effect has been considered to rely on a reflexive mechanism [55,56], being unaffected by whether a stimulus depicted a human or a humanoid robot [57].

In contrast to the accounts postulating that gaze cueing is a reflexive mechanism [55,56], it has been suggested that attentional orienting in response to gaze direction is susceptible to top-down modulation, e.g. [58,59]. For instance, Teufel and colleagues [59] showed that information about whether an observed agent could or could not see through a pair of goggles influenced automatic components of the gaze-cueing effect. Similarly, Kawai observed gaze-cueing effects only when participants believed that a potential target was visible to the gazer [60]. Wiese, Wykowska and co-workers showed that observing a robot face as a gazer in a gaze-cueing paradigm induces joint attention, but to a smaller extent (smaller gaze-cueing effects) than observing another human. This is presumably not so much due to the physical characteristics of the face, but rather attribution of mind to the observed agent [61,62] (see also §4). Interestingly, when a sample of individuals diagnosed with autism spectrum disorder (ASD) was tested in a similar gaze-cueing paradigm [63], the pattern was reverse relative to when healthy participants were tested. That is, joint attention was induced to a larger extent (larger gaze-cueing effects) by a robot face, as compared to a human face, which is in line with previous findings that demonstrated a stronger visuomotor priming effect in children with ASD when observing a reach-to-grasp action by a robotic arm, relative to observing a human [64]. The larger joint attention effect for robot faces as compared to human faces in a sample of individuals diagnosed with ASD led to the idea that joint attention can possibly be trained in individuals diagnosed with ASD with robot-assisted therapy [65]. Kajopoulos *et al.* [65] report results speaking in favour of that idea, namely that children diagnosed with ASD improved in joint attention after several weeks of interactive games with a pet-like robot, in which the children needed to follow gaze of the robot in order to complete a task inherent to the game (i.e. naming the colour of an object towards which the robot turned its head and gazed).

In summary, the collection of results of studies in which artificial agents have been used to examine early sensory processing and the joint attention mechanism suggests that while the early sensory processes of social cognition are typically not influenced by whether an interaction partner is a natural or artificial agent, engagement in joint attention is highly modulated by various factors: beliefs about the intentional agency of the interaction partner [61,62], or individual differences and social aptitude [63,65]. Thus, in contrast to the lower-level mechanisms of sensory and motor resonance, which were activated independently of the type of observed agent, the higher in the hierarchy of cognitive processes, the more the processes are sensitive to whether the interaction partner is of the same ‘kind’ or not. One of the highest-order mechanisms of social cognition is the mentalizing process, or adopting the intentional stance. Do humans engage mentalizing processes or adopt the intentional stance towards artificial agents?

## 4. Intentional stance

In order to interact with others, we need to know what they are going to do next [66]. We predict others' behaviour through adopting the intentional stance [67]. When we adopt an intentional stance towards others, we refer to their mental states such as beliefs, desires and intentions to explain and predict their behaviour. For example, when I see my best friend extending her arm with a glass of water in my direction, I assume that she *intends* to hand me that glass of water, because she *believes* that I am thirsty and she *wants to* ease my thirst. By the same token, when I see somebody pointing to an object, I infer that they *want me to* orient my attention to the object. Intentional stance is an efficient strategy for predicting behaviour of intentional systems [67]. However, for non-intentional systems, other stances, such as the design stance, might work better. For example, when driving a car, the driver predicts that the car will reduce speed when the brake pedal is pushed. Therefore, intentional stance towards others is adopted under the assumption that the observed behaviour results from operations of the mind.

### (a) Adopting the intentional stance towards artificial agents?

Neuroimaging techniques have provided evidence for brain regions related to adopting the intentional stance: the anterior paracingulate cortex [68] as well as the medial frontal cortex, left superior-frontal gyrus and right temporo-parietal junction, among others [69–71]. Adopting the intentional stance is crucial for many cognitive and perceptual processes, even the most basic ones that are involved in social interactions. For example, Stanley *et al.* [72] observed that the belief as to whether an observed movement pattern represents human or non-human behaviour modulated interference effects related to (in)congruency of self-performed movements with observed movements. Similarly, ocular tracking of a point-light motion was influenced by a belief regarding the agency underlying the observed motion [73]. Previous research demonstrated that mentalizing, the active process of reasoning about mental states of an observed agent, influenced numerous social mechanisms including perception and attention (e.g. [59]).

An experimental paradigm designed to investigate the neural correlates associated with adopting the intentional stance [68] was adapted to assess whether such a stance was adopted when interacting with a humanoid robot [70,74]. Briefly, participants in the MRI scanner played a stone–paper–scissors game while believing they were interacting with agents differing in terms of intentional nature. In the original paradigm, participants believed they played against a fellow human, an algorithm using specific rules, or a random number generator. Importantly, brain responses were always analysed when, unbeknownst to them, participants were playing against a preprogrammed sequence, so that only their belief about the intentional nature of the other agent affected physiological changes. Interacting with an intentional agent compared with a computer was associated with activation in the medial anterior prefrontal cortex, identified as a correlate of adopting the intentional stance [68]. In more recent works the computer was replaced by a humanoid robot, and a similar medial prefrontal area was found to be more active for the human than the robot or random number generator, with no differences between the two [70], as well as in another area involved in thinking about other intentional agents, the left temporo-parietal junction. Interestingly, using a similar manipulation with another social game, the Prisoner's Dilemma, resulted in the same finding [71]: areas associated with adopting the intentional stance in the medial prefrontal and left temporo-parietal junction were not activated in response to artificial agents, whether or not they were embodied with a human-like appearance. This effect was reproduced in a sample of young adults with ASD, while differences from control were found in the subcortical hypothalamus [74]. Therefore, although robots can be used to train joint attention in children in ASD, the present results indicate that robots do not naturally induce an intentional stance in the human interacting partner either in the overall population, or in patients diagnosed with ASD.

### (b) The impact of adopting the intentional stance on joint attention

Wiese *et al.* [61] showed that joint attention is influenced by beliefs that humans hold regarding whether the behaviour of an observed agent is a result of mental operations or of only a mindless algorithm. In a gaze-cueing paradigm, pictures of human or robot faces were presented. Gaze-cueing effects were larger for the human faces, as compared to robot faces. However, the effect was not related to the physical characteristics of the faces, because in two follow-up studies, the authors showed that mere belief about intentional agency of the observed gazer (manipulated via instruction) influenced the gaze-cueing effects, independently of the physical appearance of the gazer. That is, when a robot's gaze behaviour was believed to be controlled by another human, gaze-cueing effects were as large as for the human face. By contrast, when the human face was believed to represent only a mannequin, gaze-cueing effects were at the equivalent level to the robot face. In a follow-up study, Wykowska *et al.* [62] investigated the neural correlates of this behavioural effect with ERPs of an EEG signal. The findings indicated that early attention mechanisms were sensitive to adoption of the intentional stance. That is, the P1 component of the EEG signal observed at the parieto-occipital sites, within the time window of 100–140 ms was more positive for validly versus invalidly cued targets in the condition in which participants believed that the gazer's behaviour was controlled by a human. This effect was not observed in the condition in which participants were led to believe that the gazer's behaviour was pre-programmed. This provided strong support for the idea that very fundamental mechanisms involved in social cognition are influenced when adopting the intentional stance.

The authors proposed the Intentional Stance Model of social attention [62]. According to the model, higher-order social cognition, such as adopting the intentional stance towards an agent influences the sensory gain mechanism [75] through parietal attentional mechanisms. In other words, adopting the intentional stance biases attention, which in turn biases the way sensory information is processed. In that sense, higher-order cognition has far-reaching consequences for earlier stages of processing, all the way down to the level of sensory processing.

In sum, both neuroimaging as well as behavioural studies suggest that higher-order social cognition, mentalizing, and adopting the intentional stance in particular, are influenced by whether humans interact with or observe natural agents versus artificial agents. Importantly, it is not necessarily the physical appearance of an agent that plays a role in these mechanisms, but often mere belief regarding its nature. Future research will need to systematically compare the effect of actual presence of an embodied robot to experimental protocols in which embodied agents are presented on the screen as pictures or videos. According to Schilbach *et al.* [1], the actual embodied presence should evoke the mechanisms of social cognition in humans more naturally (and more similarly to natural human–human interaction) as compared to stimuli observed on the screen. However, other parameters could play a more substantial role in evoking mechanisms of social cognition, such as contingency of behaviour of the observed agent upon the behaviour of the observer [1].

As higher-order social cognitive processes are influenced by whether an agent is believed to be of a natural kind or artificial, this belief has an impact on how natural social interaction with artificial agents will be. As appearance itself is not the key factor in mentalizing or adopting the intentional stance, it is perhaps possible to imitate human-like behaviour in artificial agents, and thereby make mentalizing or adopting the intentional stance towards the artificial agents more likely. Before one can take such an approach, it is important to answer the question of whether the human brain is actually sensitive to the subtle behavioural characteristics of an agent.

## 5. Sensitivity to human-like behaviour

Perceiving others as of ‘natural’ or ‘artificial’ kind might be related to subtle characteristics of their behaviour. Whether the human brain has sensitivity for human-like behavioural characteristics of others is intriguing given the rise of artificial agents, and artificial intelligence in general. The question of what are the unique human characteristics has been addressed by philosophers with different perspectives on how humanness is defined. A ‘comparative view’ states that characteristics of humanness are those that separate us from other species in a category boundary [76,77]. On the contrary, a non-comparative perspective states that humanness is based on features essential to humans, but not necessarily unique for humans. Both these views point out, however, that humanness can be characterized by certain distinguishable features.

There is ample empirical evidence showing that humans are sensitive to discriminating biological from non-biological motion [78–80]. In a typical study addressing this issue, simple point-light dots are presented to participants with movement patterns modelled either after a biological or non-biological motion [79,80]. Already infants are able to discriminate biological motion, which suggests that this ability might be in-born in humans [81–83]. In the context of using robots as stimuli for studying social cognition, it is important to note that the brain's sensitivity to biological motion affects motor contagion, i.e. imitation of an observed movement pattern [46]. Here, we will focus on sensitivity to more subtle characteristics of human behaviour: predictability of action patterns and temporal variability.

### (a) Predictability of actions

Human movement patterns typically constitute a predictable sequence. According to Schubotz & von Cramon [84], each action sequence has a ‘syntax’: a basic schedule that is fixed and mandatory (though tolerating some level of flexibility). Goal-directed actions follow a largely predefined pattern: a coherent sequence of steps, which makes actions relatively predictable [84]. This allows for successful anticipation of possible future events through recognition of others' action sequences. Interestingly, as subtle characteristics of a movement differ dependent on an intention an agent has (e.g. different finger kinematics during reach-to-grasp with the intention to pour versus displace or pass), movement kinematics can allow predictions regarding what an agent is going to do next and can also be informative regarding the agent's intentions [85–87].

Inference of intentions plays a pivotal role in understanding and recognizing actions of others [66,88]. In this context, humanoid robots have been postulated to offer a unique opportunity to examine how intentions are inferred from movement patterns [4]. Some researchers [4] postulated that if a robot motor repertoire is similar to that of a human, and if a movement pattern is modelled after typical human-like movements, then it is likely that this movement will elicit the same reactions in a human as other humans would. In that context, an interesting observation was reported in [89] where the authors found that participants observing the humanoid robot iCub transporting an object, anticipated the action patterns similarly to when they observed a human. Therefore, the robot evoked automatic ‘motor matching’ and ‘goal reading’ mechanisms in the observers [4, p. 4].

### (b) Behavioural variability

Human actions are highly variable: for example, if our task was to produce a repetition of identical actions (both in terms of motor patterns and timing), we would not be able to do so. Variability in behaviour might be evolutionarily adaptive [90,91]. Evidence supports presence of an optimal state of variability for healthy and functional movement [92]. This variability has a particular organization and is characterized by a chaotic structure. Deviations from this state can lead to biological systems that are either overly rigid, or noisy and unstable. Both extremes lead to less adaptability to perturbations, as in the case of unhealthy pathological states or absence of skilfulness.

Wykowska *et al.* [93,94] examined how much sensitivity the human brain has for subtle (human-like) temporal variability in Turing test scenarios involving humanoid robots. In several studies, participants were seated opposite to an embodied robot. The robot was programmed to point to [93] or to gaze [94] towards a stimulus on a screen. In one condition, the onset of the pointing/gazing movement was programmed and set to a fixed temporal delay relative to the beginning of an experimental trial. In another condition, this delay was given either by an actual key press of an experimenter seated in a different room [93], or was based on pre-recorded key press times of a human [94]. Participants had to discriminate the ‘human-controlled’ from ‘programmed’ conditions, and were not instructed with regard to the hint they should use. The results showed that participants had above-chance sensitivity to human-like behaviour, although they were not aware of the hints on which they based their judgement. Hence, the human brain is sensitive to subtle characteristics of human-like behaviour, although this sensitivity might be implicit (i.e. not reaching the conscious awareness) and is related to a general individual social aptitude [94].

As the results described in this paragraph suggest that the human brain has sensitivity to human-like characteristics of behaviour, it might make sense to implement such behaviours in robots to make them appear more human-like. A more human-like behaviour might affect higher-order social cognition in such a way that artificial agents will be treated similarly to other ‘natural’ agents, which will then affect lower-level mechanisms of social cognition. In end effect, through an appropriate design of their behaviour, artificial agents might be made to elicit mechanisms of social cognition similar to those of other humans. Whether this is a desired outcome remains to be answered, taking into account ethical considerations. Do we want to aim for artificial agents to be treated as social interaction partners of the same kind as other humans? This question falls outside of the scope of this paper, but is an important one to raise for future debate.

## 6. Conclusion

To conclude, we postulate that using artificial agents (and embodied humanoid robots in particular) to examine social cognition offers a unique opportunity for combining a high degree of experimental control on the one hand, and ecological validity on the other. The state-of-the-art research which has been conducted with the use of artificial agents has uniquely informed the social cognition community about several phenomena of the human social cognition: (i) low-level processing of social visual information, including motor resonance, is preserved when artificial agents are observed instead of natural humans; (ii) by contrast, higher-order social cognitive processes are influenced by whether an agent is of ‘natural’ or ‘artificial’ kind; (iii) higher-order assumptions that humans have regarding the agents with whom they interact have profound consequences for even most fundamental processes of sensing and perception in social contexts; (iv) humans are highly sensitive, although often at the implicit level, to subtle characteristics of appearance and behaviour that indicate humanness. Therefore, ‘emulating’ human-like behaviour in artificial agents might lead to social cognitive mechanisms being invoked to the same extent as other human interaction partners would do. In sum, we propose that agents should be considered *social* when they can evoke mechanisms of social cognition in humans to the same extent as other humans do during interaction. This entails that social cognitive neuroscience methods involving interaction protocols with humanoid robots should be the preferred avenue taken when the aim is to provide artificial agents with features that increase their social competence.
